# Detection of Circovirus in Foxes with Meningoencephalitis, United Kingdom, 2009–2013

**DOI:** 10.3201/eid2107.150228

**Published:** 2015-07

**Authors:** Steve Bexton, Lidewij C. Wiersma, Sarah Getu, Peter R. van Run, Georges M.G.M. Verjans, Debby Schipper, Claudia M.E. Schapendonk, Rogier Bodewes, Lucy Oldroyd, Bart L. Haagmans, Marion M.P. Koopmans, Saskia L. Smits

**Affiliations:** RSPCA Norfolk Wildlife Hospital, East Winch, United Kingdom (S. Bexton);; Erasmus Medical Center, Rotterdam, the Netherlands (L.C. Wiersma, S. Getu, P.R. van Run, G.M.G.M. Verjans, D. Schipper, C.M.E. Schapendonk, R. Bodewes, B.L. Haagmans, M.M.P. Koopmans, S.L. Smits);; Abbey Veterinary Services, Newton Abbot, United Kingdom (L. Oldroyd);; National Institute for Public Health and the Environment, Bilthoven, the Netherlands (M.M.P. Koopmans)

**Keywords:** encephalitis, foxes, meningoencephalitis, viruses, circovirus, random amplification, brain, cerebrospinal fluid, serum, cyclovirus, neurologic disease, United Kingdom

## Abstract

A fox circovirus was identified in serum samples from foxes with unexplained neurologic signs by using viral metagenomics. Fox circovirus nucleic acid was localized in histological lesions of the cerebrum by in situ hybridization. Viruses from the family *Circoviridae* may have neurologic tropism more commonly than previously anticipated.

Circoviruses (family *Circoviridae*) are nonenveloped, single-stranded, circular DNA (≈2 kb) viruses (*1*). Two genera, *Circovirus* and *Gyrovirus*, are recognized, and an additional genus, *Cyclovirus*, has been proposed (*1*,*2*). Circoviruses have an ambisense genome organization with 2 major inversely arranged open reading frames encoding the rolling circle replication initiator protein gene (Rep) and a capsid protein gene (Cap) (*1*). A conserved stem–loop structure, required for viral replication, is located between the 5′ ends of the 2 main open reading frames. Circoviruses are thought to exhibit host species specificity and have been detected in various species, including birds, pigs, and dogs (*1*,*3*,*4*). These viruses have been associated with a variety of diseases, including respiratory and enteric disease, dermatitis, and reproductive problems (*1*,*3*–*5*). Recently, many small circular DNA genomes have been described from different hosts by using different methods, including high-throughput sequencing (*6*). Here we describe the identification, characterization, and prevalence of a newly discovered fox circovirus that was present in serum and brain samples from foxes with unexplained meningoencephalitis in the United Kingdom.

## The Study

During 2009–2013, a total of 31 adult foxes with signs of a neurologic disorder were brought to the RSPCA Norfolk Wildlife Hospital in East Winch, United Kingdom. The foxes exhibited abnormal behavior, lack of fear, reduced alertness, aimless wandering, circling, facial muscle twitching, hind limb paresis, and visual abnormalities. Cases were only detected when free-living foxes became debilitated and were taken to the wildlife rescue center. Once in captivity, diseased foxes had good appetite and generally survived with no substantial disease progression or death, but they showed no evidence of natural recovery. After a few weeks, the foxes were usually euthanized because they did not respond to (nonspecific) medical treatment. All procedures were performed in compliance with relevant laws and institutional guidelines. Following euthanasia, necropsies were performed according to standard procedures. Samples were stored in 10% neutral buffered formalin and embedded in paraffin, and 4 μm–thick sections were stained with hematoxylin and eosin and evaluated for the presence of histologic lesions.

**Figure 1:**
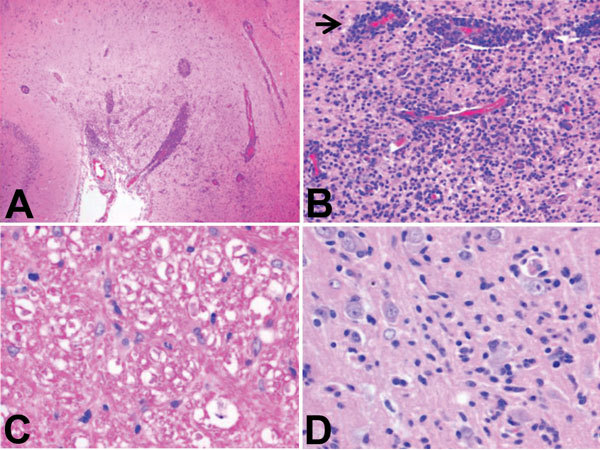
Histopathologic features of brain tissue from foxes with possible virus-induced neurologic disease. A) Multifocal, randomly distributed areas of severe encephalitis and meningitis in the cerebrum (original magnification ×40). B) Detail of encephalitis in the cerebrum (original magnification ×200). Gray and, to a lesser extent, white matter of the cerebrum showed randomly dispersed areas of astrocytosis, gliosis, and infiltration with lymphocytes and plasma cells. Blood vessels in affected areas show perivascular cuffing with distention of Virchow-Robin spaces with up to 10 layers of lymphocytes and plasma cells (arrow). C) Detail of white matter in the cerebellum (original magnification ×400). Axons in affected white matter showed degeneration, characterized by formation of spheroids, shrinkage, and fragmentation; axon sheaths containing microglia or macrophages; and presence of gitter cells in surrounding neuropil. Cerebellum was mildly affected, and meninges, especially of the cerebrum, were frequently distended with lymphocytes and plasma cells. D) Detail of gray matter of cerebrum (original magnification ×400). Individual neuronal cell bodies were frequently surrounded by up to 5 glial cells (i.e., sattelitosis) and showed margination of Nissl substance, hyperchromasia, degeneration, and necrosis. Tissue sections were subjected to conventional hematoxylin and eosin staining.

All foxes had similar histologic findings consisting of chronic multifocal or diffuse lymphoplasmacytic meningoencephalitis oriented on the forebrain with a predilection for cortical gray matter (Figure 1; Table 1; Technical Appendix Figure 1). Characteristic histopathologic features were nonspecific perivascular cuffing, rod cell proliferation, spongiosis, neuronal necrosis, moderate to severe gliosis, neuronal satellitosis, and neurophagia. Substantial pathologic changes were restricted to the central nervous system. Histopathologic changes suggested viral, protozoal, microsporidial, immune-mediated, or idiopathic disease. Immunohistochemistry of brain samples was negative for canine distemper virus, canine adenovirus, Borna disease virus, *Toxoplasma gondii*, and *Neospora canium* (data not shown). Serologic test results for canine distemper virus, rabies virus, *N. canium*, and tickborne encephalitis virus were negative, and Ziehl-Neelsen and Giemsa staining results for microsporidia were negative. Minor white matter involvement, the duration of animal survival, and the current absence of documented rabies cases in the United Kingdom eliminated rabies virus as the cause of the neurologic disorder.

**Table 1:** Overview of testing results for fox serum samples used in a study of the detection of circovirus in foxes with meningoencephalitis, United Kingdom, 2009–2013*

Animal	Year serum sample obtained	Age	Sex	County	Signs†	Outcome	454‡	PCR§	C_t_	FFPE
VS7100001	2013	Adult	F	Nor	Yes	Euthanized	Yes	Pos	16.5	Yes
VS7100002	2013	Adult	F	Ess	Yes	Euthanized	Yes	Pos	39.3	No
VS7100003	2013	Adult	F	Nor	Yes	Euthanized	Yes	Pos	16.4	Yes
VS7100004	2013	Adult	M	Suf	Yes	Released	Yes	Pos	35.0	No
VS7100005	2013	Adult	F	Nor	Yes	Euthanized	Yes	Pos	16.4	Yes
VS7100006	2013	Adult	M	Bed	Yes	Euthanized	Yes	Pos	36.9	No
VS7100010	2009	Adult	F	Lei	Yes	Euthanized	No	Pos	23.4	No
VS7100014	2010	Adult	M	Suf	Yes	Euthanized	No	Pos	14.1	Yes
VS7100015	2010	Adult	M	Lin	Yes	Euthanized	No	Pos	14.3	No
VS7100017	2011	Adult	M	Nor	Yes	Euthanized	No	Neg	ND	No
VS7100018	2011	Adult	F	Nor	Yes	Euthanized	No	Neg	ND	No
VS7100019	2011	Adult	F	Cam	Yes	Euthanized	No	Pos	36.8	No
VS7100021	2011	Adult	M	Cam	Yes	Euthanized	No	Pos	25.3	No
VS7100025	2012	Adult	M	Suf	Yes	Euthanized	No	Neg	ND	No
VS7100030	2012	Adult	F	Nor	Yes	Euthanized	No	Neg	ND	No
VS7100032	2012	Adult	M	Ess	Yes	Euthanized	No	Pos	38.0	No
VS7100038	2013	Adult	F	Suf	Yes	Euthanized	No	Pos	17.9	Yes
VS7100008	2007	Adult	M	Cam	No	Euthanized	No	Neg	ND	No
VS7100011	2009	Adult	M	Lin	No	Euthanized	No	Pos	29.7	No
VS7100012	2010	Adult	M	Nor	No	Died	No	Neg	ND	Yes
VS7100020	2011	Adult	M	Cam	No	Euthanized	No	Neg	ND	No
VS7100022	2012	Adult	M	Cam	No	Euthanized	No	Pos	39.5	No
VS7100023	2012	Adult	M	Cam	No	Died	No	Pos	37.3	No
VS7100024	2012	Adult	F	Lin	No	Euthanized	No	Neg	ND	No
VS7100026	2012	Juvenile	F	Lei	No	Released	No	Pos	14.4	No
VS7100027	2012	Juvenile	F	Nor	No	Released	No	Pos	14.6	No
VS7100028	2012	Juvenile	F	Suf	No	Released	No	Neg	ND	No
VS7100029	2012	Juvenile	F	Lin	No	Released	No	Pos	14.2	No
VS7100031	2012	Juvenile	M	Suf	No	Released	No	Neg	ND	No
VS7100033	2012	Juvenile	M	Nor	No	Euthanized	No	Neg	ND	No
VS7100035	2013	Juvenile	F	Cam	No	Released	No	Neg	ND	No
VS7100036	2013	Adult	M	Cam	No	Released	No	Pos	39.2	No

*Bed, Bedfordshire; Cam, Cambridgeshire; C_t_, cycle threshold values of real-time PCR; Ess, Essex; FFPE, formalin-fixed paraffin-embedded tissue; Lei, Leicester; Lin, Lincolnshire; ND, not determined; Neg, negative; Nor, Norfolk; Pos, positive; Suf, Suffolk. †Neurologic signs were abnormal behavior, lack of fear, reduced alertness, aimless wandering, circling, facial muscle twitching, progressive weakness of hind legs, and visual abnormalities. ‡Samples were analyzed by using a viral metagenomics approach with the 454 sequence platform (GS Junior; Roche, Basel, Switzerland). §TaqMan real-time PCR.

Serum samples from 6 of the foxes (VS7100001–6) were available for virus discovery studies. To perform the studies, we used a viral metagenomics approach with the 454 sequence platform (GS Junior; Roche, Basel, Switzerland) as described previously (*7*–*10*) (Table 1). More than 22,000 reads were analyzed as described previously (*10*–*12*) (Technical Appendix Figure 2). The complete genome sequences of circoviruses from 3 foxes were obtained; the sequences were 99% identical at the nucleotide level (GenBank accession nos. KP260925–7). The fox circovirus genomes had an ambisense organization characteristic of circovirus (Technical Appendix Figure 3). Phylogenetic analysis revealed that the genomes were closely related to those of the recently described canine circoviruses (*3*,*13*), displaying ≈92% amino acid identity in the Rep protein and ≈89% nt sequence identity across the entire genome (Technical Appendix Figure 4). On the basis of the suggested criteria demarcating species (*1*), the fox and canine circoviruses belong to the same species.

**Figure 2:**
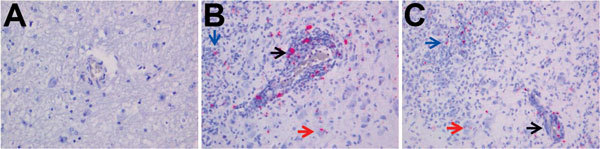
Detection of fox circovirus–specific transcripts in brain tissue of foxes with neurologic disease showing in situ hybridization of cerebrum with fox circovirus replication initiator protein gene–specific probe (original magnification ×200). A) Negative control fox VS7100012. The serum sample from this fox was negative for circovirus, and the animal did not exhibit signs of neurologic disease. B, C) Affected foxes VS7100005 and VS7100003, respectively. Both animals had neurologic disease, and their serum samples were positive for fox circovirus (see Table 1 for more information regarding these foxes). Black arrows indicate mononuclear cells in perivascular cuffs, blue arrows show inflammatory infiltrates in the neuropil, and red arrows point to staining in neuronal somata in cerebral gray matter of circovirus–positive animals with neurologic disease.

A diagnostic real-time fox circovirus PCR was performed targeting the Rep-coding sequence on 32 serum samples from foxes with and without neurologic signs (Table 1). Viral nucleic acid was extracted by using the MagNA Pure LC Total Nucleic Acid Isolation Kit (Roche, Indianapolis, IN, USA) and amplified by real-time PCR by using primers VS756 (5′-TCCGAGATAGCC GGCGTGGTA-3′), VS757 (5′-CCCGGCCACAGATCAAGTACTTA-3′), and VS758 (5′-FAM-ATCCAACTCCGGAGGAGGAGGA-TAMRA-3′) and the TaqMan Universal Master Mix (Applied Biosystems, Foster City, CA, USA). In addition to samples VS7100001–6, another 14 fox serum samples were positive for fox circovirus, indicating that the virus had infected foxes in multiple counties in the United Kingdom during past years (Table 1; Technical Appendix Figure 5). Clinical data indicated that 77% of circovirus-positive foxes had signs of neurologic disease, compared with only 47% of circovirus-negative foxes (Table 2). Fox circovirus was present in male and female foxes and in adults and juveniles (Table 2). In addition, fox circovirus was detected by real-time PCR in brain samples of 2 of 4 foxes with neurologic disease (VS7100017 and 19; cycle threshold value >35) but not in the brain tissues of 2 foxes without disease. The detection of fox circovirus nucleic acid in the cerebrum of foxes with neurologic disease was confirmed by using the RNAscope 2.0 in situ hybridization kit (Advanced Cell Diagnostics, Hayward, CA, USA) and a Rep gene–specific probe according to the manufacturer’s instructions. Negative controls consisted of circovirus-negative foxes without histopathologic disease. Multifocal fox circovirus RNA signal was detected and associated with the aforementioned histologic lesions in the cerebrum (Figure 2). Specifically, RNA signal was detected in mononuclear cells in perivascular cuffs, inflammatory infiltrates in the neuropil, and neuronal somata in cerebral gray matter of circovirus-positive foxes with neurologic disease. No circovirus signal was found in control foxes with lymphocytic cuffs due to other (known) viral infections or in control foxes without neurologic disease (Figure 2).

**Table 2:** Univariate statistical analysis of age, sex, disease signs, and circovirus real-time PCR results for foxes in a study of the detection of circovirus in foxes with meningoencephalitis, United Kingdom, 2009–2013*

Parameter	PCR results for foxes, no. (%)†		p value by χ^2^ test	OR (95% CI)
	Without neurologic signs	With neurologic signs		
All foxes	15 (46.9)	17 (53.1)		
Sex				
M	9 (60.0)	8 (47.1)	0.502	1.69 (0.41–6.88)
F	6 (40.0)	9 (52.9)		
Age, y				
Juvenile	7 (46.7)	0 (0)	0.002	3.13 (1.77–5.53)
Adult	8 (53.3)	17 (100)		
Circovirus positive				
No	8 (53.3)	4 (23.5)	0.144	3.71 (0.82–16.84)
Yes	7 (46.7)	13 (76.5)		

*OR, odds ratio. †Neurologic signs were abnormal behavior, lack of fear, reduced alertness, aimless wandering, circling, facial muscle twitching, progressive weakness of hind legs, and visual abnormalities.

## Conclusions

Our findings indicate that circoviruses commonly cause systemic infections in wild foxes in the United Kingdom and can be detected in the brains of foxes with neurologic disease. It has been suggested that circoviruses are involved in a plethora of diseases in pigs, dogs, and birds (*1*,*3*–*5*). The canine circovirus may be associated with development of vasculitis in dogs (*3*), and an overall virus prevalence in serum samples of ≈3% has been reported (*3*,*13*). However, we found that the prevalence of fox circovirus in serum samples from foxes with and without neurologic disease was much higher and more comparable to the prevalence of porcine circoviruses among pigs (*14*). No association of virus infection with vasculitis was apparent. Instead, fox circoviruses may be associated with development of neurologic disease directly or as a contributory complicating cofactor. 

Cycloviruses, which belong to a proposed new genus in the family *Circoviridae*, were recently found in serum and cerebrospinal fluid of humans with paraplegia and acute infections of the central nervous system (*11*,*15*), suggesting that viruses from the family *Circoviridae* may have neurologic tropism more commonly than previously anticipated. However, a causal link between circovirus infection and disease in humans and animals remains to be proven. Because the prevalence of circoviruses in foxes was relatively high and closely related circovirus species seem pathogenic for both dogs and foxes, additional surveillance is warranted to clarify the epidemiology and pathogenicity of circoviruses in foxes.

Technical Appendix Histopathologic features of brain tissues from foxes with possible circovirus-induced neurologic disease and relative abundance of broad taxonomic categories in metagenomic sequences obtained from fox serum samples.
