# Supplementary material for: Detection of Circovirus in Foxes with Meningoencephalitis, United Kingdom, 2009–2013
Source: Emerg Infect Dis. 2015 Jul;21(7):1205–8. doi: 10.3201/eid2107.150228 (PMC4480402; doi:10.3201/eid2107.150228)
Supplement: Technical Appendix — Histopathologic features of brain tissues from foxes with possible circovirus-induced neurologic disease and relative abundance of broad taxonomic categories in metagenomic sequences obtained from fox serum samples. [file 15-0228-Techapp-s1.pdf]

Article DOI: http://dx.doi.org/10.3201/eid2107.150228

Detection of Circovirus in Foxes with Meningoencephalitis, United Kingdom, 2009-2013

Technical Appendix

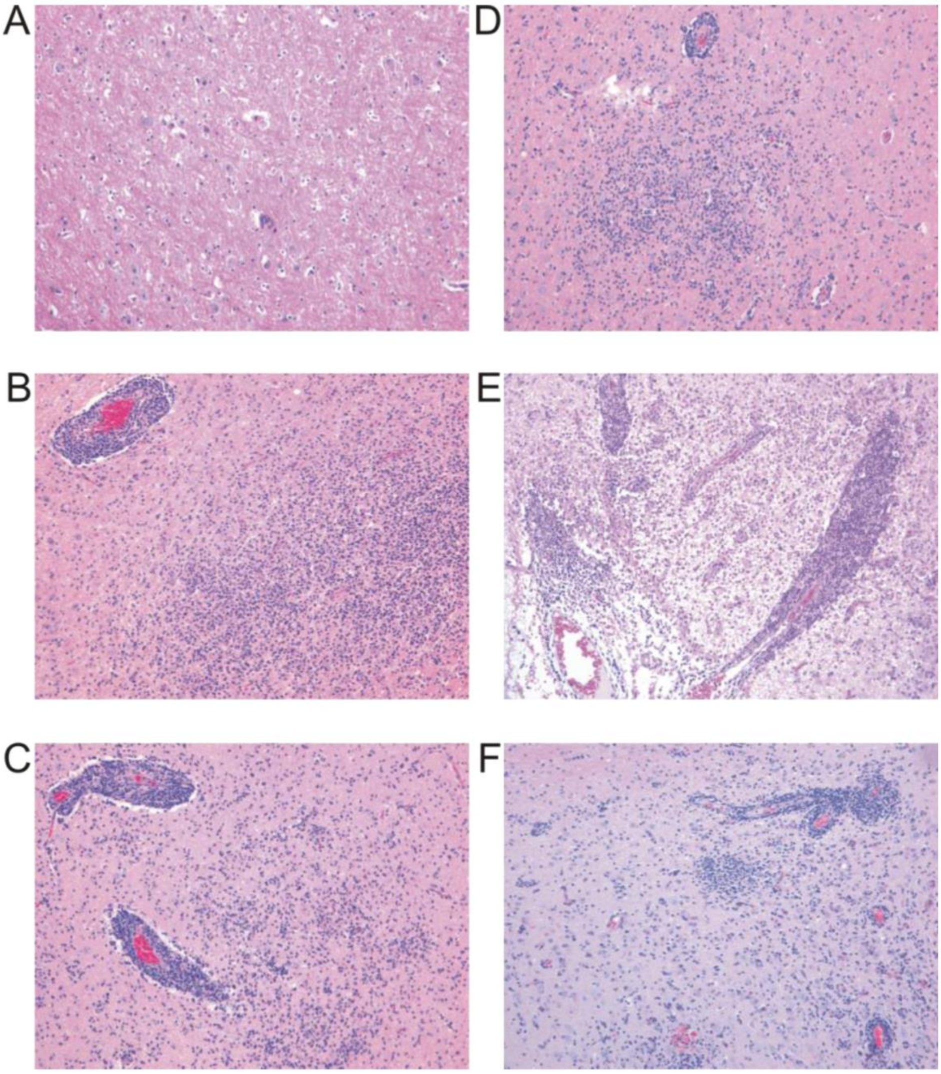

Technical Appendix Figure 1. Histopathology of brain tissues of foxes with potential virus-induced neurologic disease. A) Control animal VS7100012 negative for fox circovirus by real time PCR in serum. B–F) Affected animals VS7100005 (B), VS7100003 (C), VS7100001 (D), VS7100014 (E), and VS7100038 (F), positive for fox circovirus by real time PCR in serum showing severe encephalitis and perivascular cuffing. See Table 1 in the article text for reference to animals. Tissue sections were subjected to conventional hematoxylin and eosin staining. Original magnification ×100.

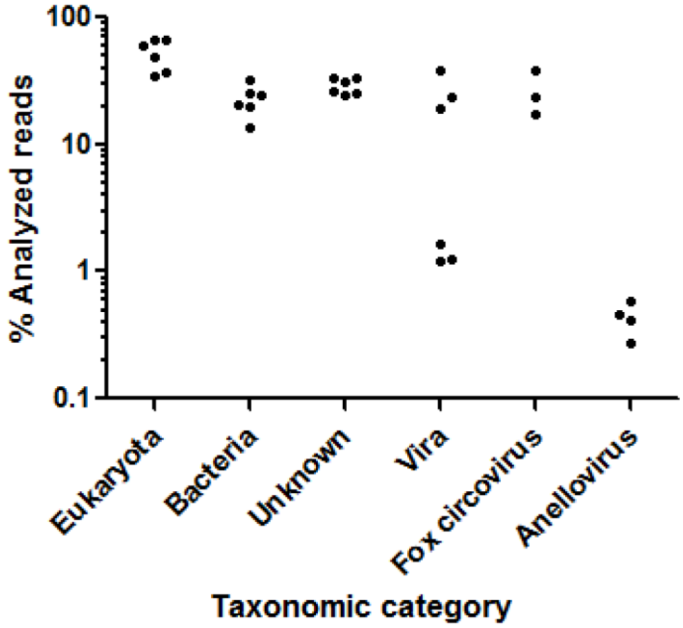

Technical Appendix Figure 2. Relative abundance of broad taxonomic categories in metagenomic sequences obtained from fox serum samples. Exhaustive iterative assembly of sequences is part of a virus discovery pipeline written in the Python 2.7 programming language, which includes trimming of reads and initial assembly with Newbler version 2.7, the Roche 454 GS De Novo Assembler software (454 Life Sciences, Branford, CT, USA), with standard parameters. Trimmed reads and initial contigs were subjected to assembly by CAP3 (version date December 21, 2007) with standard parameters. The resulting singletons and contigs were iteratively assembled by CAP3 until no new contigs were formed. Subsequently, the trimmed reads were mapped back to the identified taxonomic units with Newbler version 2.7 by using a minimum length of 75 nucleotides and standard parameters. The resulting contigs and singletons were filtered with DustMasker, which is part of the National Center for Biotechnology Information's BLAST 2.2.25 suite of tools for sequences that contain <60% low-complexity sequences. After filtering the low-complexity sequences, the remaining taxonomic units were subjected to a BLASTN search against a database that contained only nucleotide sequences from birds (*Aves*, taxonomic identification [taxID] 8782), carnivores (*Carnivora*, taxID 33554), primates (*Primates*, taxID 9443), rodents (*Rodentia*, taxID 9989), and ruminants (*Ruminantia*, taxID 9845) with an E value cutoff of 0.001 for the subtraction of potential host sequences. Sequences without hits in the host BLAST were then subjected to a BLASTN search against the entire nucleotide database with an E value cutoff of 0.001. Due to limited capacity, all the sequences without hits were then subjected to a BLASTX search against sequences present in the GenBank nr database with an E value cutoff value of 0.001. BLAST hits were categorized by assigning taxonomic categories. Archeal sequences or sequences with an unidentified taxonomic category assigned were excluded (these include, for example, human artificial chromosome vectors, for which no taxonomic category could be defined). Most viral sequences that were not fox circovirus or anellovirus were from bacteriophages and, to a lesser extent, large DNA viruses.

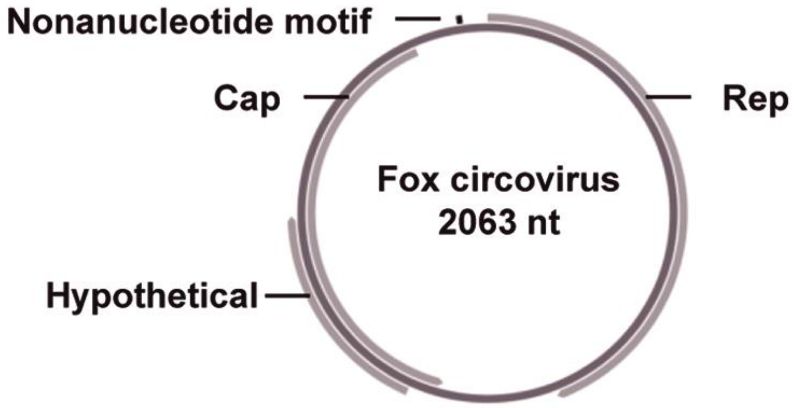

Technical Appendix Figure 3. Schematic representation of the fox circovirus genome. Replication initiator protein gene (Rep) and capsid protein gene (Cap) are indicated.

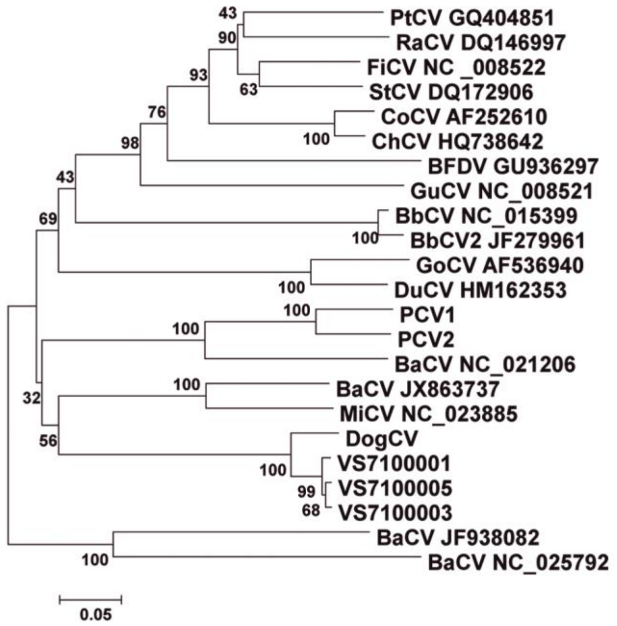

Technical Appendix Figure 4. Phylogenetic tree of the replication initiator protein gene amino acid sequences of fox circoviruses VS7100001, VS7100003, and VS7100005 and representative other circoviruses. The tree was generated by using MEGA5 (http://www.megasoftware.net/) with the neighbor-joining method with p-distance and 1,000 bootstrap replicates. Bootstrap values >50% are shown at tree nodes. Scale bar represents 5% estimated phylogenetic divergence. GenBank accession numbers are indicated. PtCV, chimpanzee stool avian-like circovirus Chimp17; RaCV, raven circovirus; FiCV, finch circovirus; StCV, starling circovirus; CoCV; columbid circovirus; ChCV chicken circovirus; BFDV, beak and feather disease virus; GuCV, gull circovirus; BbCV, barbel circovirus; GoCV, goose circovirus; DuCV; duck circovirus; PCV, porcine circovirus; BaCV, bat circovirus; MiCV, mink circovirus; DogCV, dog circovirus.

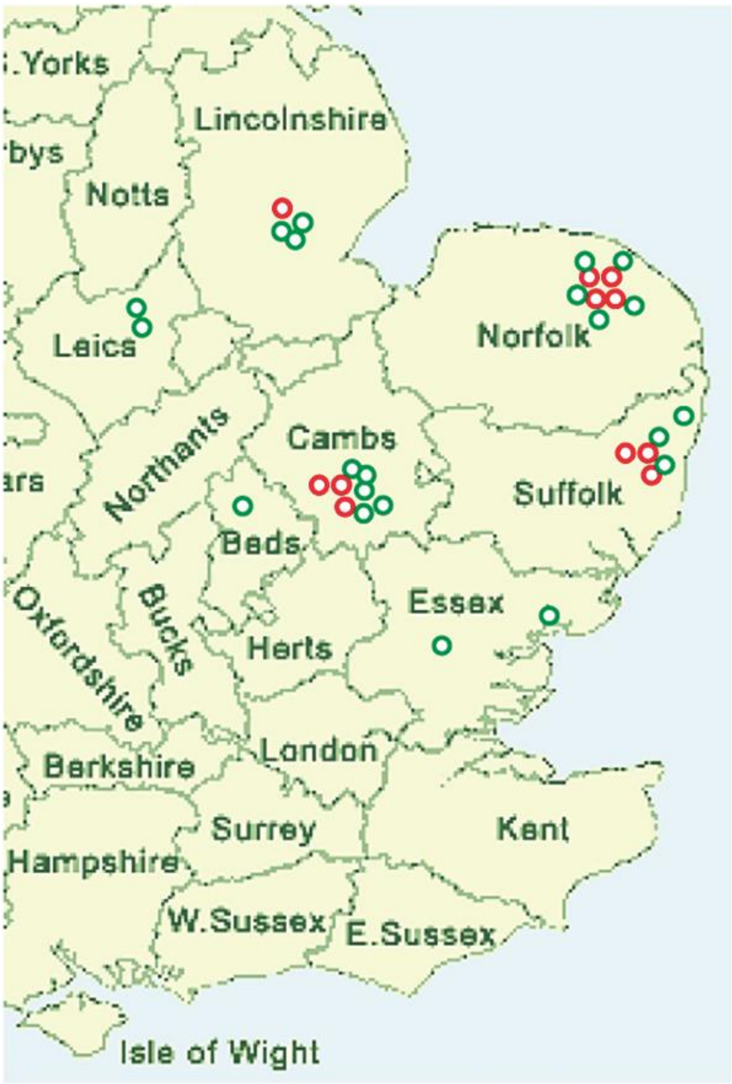

Technical Appendix Figure 5. Geographic distribution of fox circovirus–positive (green circles) and –negative (red circles) foxes in the United Kingdom. Virus positivity was determined by real-time PCR.
